# Pro-apoptotic Action of Corticosterone in Hippocampal Organotypic Cultures

**DOI:** 10.1007/s12640-016-9630-8

**Published:** 2016-05-17

**Authors:** Anna Kurek, Mateusz Kucharczyk, Jan Detka, Joanna Ślusarczyk, Ewa Trojan, Katarzyna Głombik, Bartosz Bojarski, Agnieszka Ludwikowska, Władysław Lasoń, Bogusława Budziszewska

**Affiliations:** Department of Experimental Neuroendocrinology, Institute of Pharmacology, Polish Academy of Sciences, 12 Smętna Street, 31-343 Kraków, Poland; Department of Veterinary Science, Faculty of Animal Science, University of Agriculture, 24/28 Mickiewicza Street, 30-059 Kraków, Poland; Department of Neuroscience, Physiology and Pharmacology, University College London, Gower St, London, WC1E 6BT UK

**Keywords:** Corticosterone, Glutamate, Apoptosis, Hippocampus, Organotypic cultures

## Abstract

Elevated levels of glucocorticoids exert neurotoxic effects, and the hippocampus is particularly sensitive to the effects of glucocorticoids. Because some data have indicated that an increased action of glucocorticoids in the perinatal period enhances the susceptibility of brain tissue to adverse substances later in life, the main purpose of the present study was to compare necrotic/apoptotic corticosterone action in hippocampal organotypic cultures obtained from control animals with the effect of this steroid in tissue from prenatally stressed rats. Because the adverse effects of glucocorticoid action on nerve cell viability appear to result mainly from an increase in the intensity of the effects of glutamate and changes in growth factor and pro-inflammatory cytokine synthesis, the involvement of these factors in corticosterone action were also determined. In stress-like concentration (1 μM), corticosterone, when added to hippocampal cultures for 1 and 3 days, alone or jointly with glutamate, did not induce necrosis. In contrast, in 3-day cultures, corticosterone (1 μM) increased caspase-3 activity and the mRNA expression of the pro-apoptotic *Bax*. Moreover, corticosterone’s effect on caspase-3 activity was stronger in hippocampal cultures from prenatally stressed compared to control rats. Additionally, 24 h of exposure to corticosterone and glutamate, when applied separately and together, increased *Bdnf*, *Ngf*, and *Tnf-α* expression. In contrast, after 72 h, a strong decrease in the expression of both growth factors was observed, while the expression of TNF-α remained high. The present study showed that in stress-like concentrations, corticosterone exerted pro-apoptotic but not necrotic effects in hippocampal organotypic cultures. Prenatal stress increased the pro-apoptotic effects of corticosterone. Increased synthesis of the pro-inflammatory cytokine TNF-α may be connected with the adverse effects of corticosterone on brain cell viability.

**Electronic supplementary material:**

The online version of this article (doi:10.1007/s12640-016-9630-8) contains supplementary material, which is available to authorized users.

## Introduction

Glucocorticoids easily cross the blood–brain barrier and bind to intracellular mineralocorticoid (MR) and glucocorticoid (GR) receptors in neuronal and glial cells. Glucocorticoids are essential for the maintenance of homeostasis and adaptations to stress; however, their long-term, excessive release leads to damage of different brain structures, particularly the hippocampus. The hippocampus is one of the most sensitive regions of the brain to neurotoxic factors. This structure plays a significant role in learning and memory processes (Eichenbaum et al. 1992; De Kloet et al. 1999; Hui et al. 2004); therefore, its injury disturbs vital functions of the body. It is well described that prolonged stress or long-term glucocorticoid treatment causes neuronal loss in the hippocampus (especially in the CA3 region) in rats and primates (McEwen 1999; Sapolsky et al. 1985, 1990), evokes reorganization of dendrites in hippocampal area CA3, reduces branch density and the number of synapses, and inhibits neurogenesis (Duman 2002; Magarinos et al. 1999; Sousa et al. 2000). However, the neurotoxic mechanism of glucocorticoid action is poorly understood. It is known that increased glucocorticoid concentrations can enhance synaptic glutamate concentrations, affect the synthesis and action of pro-inflammatory cytokines and attenuate the synthesis of brain-derived neurotrophic factor (BDNF). All of these changes may be responsible for nerve cell damage and also seem to be involved in the pathogenesis of affective disorders.

It is known that the effects of glucocorticoids depend not only on their concentration and duration of action but also on the period of life in which an organism is exposed to them. In adult animals, these hormones usually evoke short-lasting changes, while glucocorticoid exposure in the perinatal period permanently changes the expression of some genes and, as a consequence, causes long-lasting disturbances in the levels of neurotransmitters, their receptors, hypothalamic–pituitary–adrenal (HPA) axis activity, and GR regulatory factors (Matthews 2000; Szymańska et al. 2009). Moreover, glucocorticoids acting in the perinatal period can increase tissue susceptibility to the adverse effects of lipopolysaccharide, stress, and glutamate in adulthood (Diz-Chaves et al. 2013; Kohman et al. 2008; Szczęsny et al. 2014). Furthermore, prenatal stress has been shown to weaken neurogenesis-related processes in the dentate gyrus of the hippocampus and to lower cell survival in young and adult animals (Lemaire et al. 2006). We previously showed that prenatal stress enhanced the effect of immobilization stress or peripheral glucose administration on brain glucose, glycogen, corticosterone, and glucose transporter concentrations in later life (Detka et al. 2014, 2015).

The existing data on the participation of glucocorticoids in neurotoxicity and the pathogenesis of depression indicate that an increase in the intensity of glutamate action, most likely through inhibition of its uptake by astrocytes and elevation of its extracellular level, is the most substantial mechanism underlying the effects of glucocorticoids. This hypothesis is supported by data showing that increased glucocorticoid or glutamate levels induce similar changes in the apical dendrites of pyramidal cells in hippocampal area CA3 and that the administration of an antagonist of excitatory amino acid receptors prevented these changes (Magariños and McEwen 1995).

However, in contrast to in vivo studies, the majority of in vitro experiments have not produced unequivocal results and documented the neurotoxic potential of glucocorticoids only when they were used at very high concentrations. Although the studies conducted by Zhu et al. (2006) indicated that exposing hippocampal neurons to 1 µM corticosterone (similar to the concentration used to elicit a strong stress reaction) led to neuronal damage; however, other studies suggested that this concentration did not cause cell damage (Sapolsky et al. 1988; Roy and Sapolsky 2003). It was also shown that neuronal cultures were more vulnerable to potentially neurotoxic corticosterone effects when they were cocultured with glia (Dugan et al. 1995; Lewis et al. 1998). Because in vivo studies have demonstrated the effect of glucocorticoids on many nervous system cells (neurons, astrocytes, and microglia), it appears that the lack of junctions and absence of interactions between different cells may be the cause of the weak cytotoxic effect of glucocorticoids observed in the in vitro studies, which were most often conducted on neuronal cultures. Out of all in vitro models, organotypic cultures, which contain all types of nervous system cells and their natural connections, are the closest to in vivo conditions. However, the few existing studies of organotypic cultures utilized only cultures initiated from tissues derived from control animals, while HPA axis activity and the strength of glucocorticoid actions in adult animals largely depends on factors present in the perinatal period.

The aim of the present study was to determine: (1) whether corticosterone in a concentration comparable to its level in the brain and at a level that is 10 times higher evokes necrotic or apoptotic changes in organotypic cultures of the hippocampus; (2) if the adverse corticosterone effect is stronger in tissue from prenatally stressed compared to control animals; (3) if glutamate increases the adverse effects of corticosterone; (4) if detrimental corticosterone effects are present after its presence in the culture medium for 24 h and/or 72 h; (5) if the necrotic or apoptotic effect of corticosterone is connected with changes in the expression of neurodegenerative, pro-inflammatory cytokine (tumor necrosis factor-TNF-α), or growth factors (BDNF and NGF).

## Materials and Methods

### Animals

Sprague–Dawley rats (200–250 g) were purchased from Charles River Corporation (Germany). The rats were kept in an animal housing facility at a room temperature of 22 ± 1 °C and with a 12/12 h light/dark cycle (lights on at 07.00 a.m.). Food and water were available ad libitum. All experiments were conducted in compliance with the ARRIVE guidelines and were approved by the second Local Ethics Committee in Kraków, Poland (No. 926/2012). After 1 week of acclimatization, vaginal smears were collected from females daily to determine the estrous cycle phase. During proestrus, the female rats were placed with males for 12 h. Next, the vaginal smears were tested for the presence of sperm. Then, pregnant females were randomly assigned to the control and stress groups.

### Stress Procedure

The prenatal stress procedure was performed as previously described by Morley-Fletcher et al. (2003, 2004). Pregnant rats were subjected daily to three stress sessions, starting at 09.00, 12.00, and 17.00 h. During the sessions, the rats were placed in plastic cylinders (7/12 cm) and exposed to a bright light for 45 min. The stress sessions were performed from the 14th day of pregnancy until delivery. Control pregnant females were left undisturbed in their home cages. In total, 12 pregnant female rats were included in the present study (six in the control and 6 in the stress group).

### Establishment of Organotypic Cultures

Seven-day-old male pups from both the control and stressed group were decapitated with scissors, and their brains were aseptically and quickly removed and placed in an ice-cold working buffer (96 % HBSS, 3.5 % glucose, and 0.5 % penicillin/streptomycin). The hippocampi were separated, transferred to Teflon disks and cut into 400-µm slices using a McIlwain tissue chopper. From 6 pregnant control animals 75 female and male pups were obtained while from 6 pregnant females subjected to stress 72 pups were born. All the 7-day-old pups were decapitated in order to separate hippocampi. For each experiment, two hippocampi were separated. In total, 150 hippocampi from control and 144 from prenatally stressed animals were used in eight independent cultures performed in 4 series (192 individual wells). Since only slices with the intact morphology were used, about 10–15 % of slices damaged during the cutting were rejected.

Organotypic hippocampal cultures were established according to the method of Stoppini (1991). The slices were transposed onto Millicell-CM (Millipore) membranes for further growth. The Millicell-CM membranes in 6-well plates were preequilibrated with 1 ml of culture medium (50 % DMEM; pH 7,4; 25 % HBSS; 25 % horse serum; 5 mg/ml glucose; 1 % amphotericin B; 0,4 % penicillin–streptomycin; 2 % B-27 supplement). The cultures were maintained for 7 days under standard conditions in an incubator (37 °C) with adjustable CO_2_ flow (5 %).

The cultures were initiated in a regular 25 % horse serum-containing medium, which was then gradually (from DIV 4th until 7th) replaced with a serum-free medium (50 % DMEM F-12; pH 7,4; 25 % HBSS; 5 mg/ml glucose; 1 % amphotericin B; penicillin–streptomycin; 2 % B-27 and 2 % N-2 supplements).

### Corticosterone and Glutamate-Induced Neurotoxicity

The hippocampal slices were cultured for 7 days before glutamate and/or corticosterone application. Twenty-four hours before the application, the culture medium was supplemented with propidium iodide at the lowest effective concentration (1.5 µM) to assess slice damage. The slices were examined under a fluorescence microscope (Ex/Em: 536/620).

After 7 days in culture, corticosterone and/or glutamate was added for 1–3 days. The corticosterone was dissolved in ethanol and diluted ×1000 in culture medium, while the glutamate was dissolved in culture medium. Both compounds were added to the culture medium in a volume of 10 µl. NMDA dissolved in culture medium was used as a positive control. The control cultures were supplemented with an appropriate amount of vehicle.

Experiments were conducted on the following groups: hippocampal slices from control or prenatally stressed animals that were cultured with vehicle; 1 and 10 μg/ml corticosterone; 10 and 100 μg/ml glutamate; and corticosterone and glutamate (1 + 10 μg/ml and 10 + 100 μg/ml, respectively).

### Uptake of propidium iodide

Thirty minutes before the final detection, the cultures were supplemented with propidium iodide at a concentration of 10 µM to visualize dead cells. In all cultures, hippocampal cytotoxicity (PI staining of cells with damaged membranes) was detected by fluorescence microscopy (AxioCam MRm, ZEISS, Germany). As a marker of cytotoxicity, PI staining significantly correlates with other reliable measures of cell death. PI fluorescence reflects the staining of necrotic or end-stage apoptotic cells. PI has a maximum excitation wavelength of 536 nm, and the emission of PI in the visual range is 620 nm (Kawalec et al. 2011). Images of slices were converted into BMP format with an equivalent pixel number, and these images were analyzed to estimate the degree of damage (ImageJ). The obtained results are presented as a percentage of the corresponding value in control cultures.

The percentage of dead cells (PI positive) was calculated according to the following formula: % dead PI-positive cells = (slice fluorescence intensity [*F*1] − *F* background/max [*F*1] − *F* background) × 100 %; the max [*F*1] value was obtained by exposing slices to 1 mM NMDA.

### Determination of Lactate Dehydrogenase Activity

Lactate dehydrogenase (LDH) activity in the culture medium was assayed using a colorimetric method (Cytotoxicity Detection Kit, Roche Diagnostic GmbH). The LDH activity was determined through coupled enzymatic reactions. In the first reaction, LDH reduces NAD+ to NADH+ H+ through the oxidation of lactate to pyruvate, and in the second reaction, the diaphorase transfers H/H + from NADH + H + to the tetrazolium salt INT and reduces it to colored formazan. The formazan dye that is formed is water soluble, shows a broad absorption maximum at approximately 500 nm, and can be quantified by measuring the absorbance at 400–550 nm. The increase in LDH release from membrane-damaged cells into the culture medium is correlated with the increase in the number of dead cells and the amount of formazan. The results are presented as percentage of the value obtained in control cultures, where 100 % represents the LDH activity in slices cultured only with the appropriate vehicle.

### Determination of Caspase-3 Activity

Caspase-3 activity was determined in slices of the hippocampus using a Caspase-3 Fluorometric Assay Kit (Sigma Aldrich), in which the hydrolysis of acetyl Asp-Glu-Val-Asp 7-amido-4-methylcoumarin (Ac-DEVD-AMC) by caspase 3 results in the release of fluorescent 7-amino-4-methylcoumarin (AMC). The excitation and emission wavelengths of AMC are 360 and 460 nm, respectively. The concentration of the AMC was calculated from a calibration curve prepared with AMC standards. The obtained results are presented as a percentage of the value obtained in control cultures.

### Real-Time PCR

Freshly isolated hippocampal tissue samples were immediately placed in RNALater^®^ solution (Applied Biosystems, USA) and stored either at 4 °C (if homogenization was expected to occur in less than 4 weeks) or at −20 °C prior to total RNA extraction.

### RNA Extraction and cDNA Preparation

The tissue samples were submerged in lysis buffer (Roche, Germany). Total RNA was extracted using the RNeasy Mini Kit (Qiagen, Hilden, Germany) following the manufacturer’s instructions. The RNA concentration was measured using a NanoDrop ND-1000 Spectrometer (Thermo Scientific, Wilmington, USA). Identical amounts of RNA (1 µg) were reverse transcribed into cDNA using a commercial RT-PCR kit (Applied Biosystems, USA) according to the manufacturer’s instructions.

### Quantitative PCR

The PCR was performed using TaqMan probes and primers for the genes encoding *Tnf*-*α* (Rn99999017_m1), *Bdnf* (Rn02531967), *Ngf* (Rn01533872_m1), and *Bax* (Rn02532082_g1) (Applied Biosystems, Foster, CA, USA) and the FastStart Universal Probe Master (Rox) kit (Roche Diagnostic, Germany). Amplification was performed in a total volume of 20 µl of mixture, which contained 1× FastStart Universal Probe Master (Rox) mix (Roche, Germany), 50 ng of cDNA used as the PCR template, 900 nM TaqMan forward and reverse primers, and a 250 nM hydrolysis probe labeled with the fluorescent reporter dye FAM at the 5′-end and with a quenching dye at the 3′-end. The thermal cycling conditions were as follows: 2 min at 50 °C and 10 min at 95 °C, followed by 40 cycles at 95 °C for 15 s and 1 min at 60 °C. The samples were run in duplicate (CFX96 Real-Time System, BIO-RAD, Hercules, CA, USA). The threshold value (*C*_t_) for each sample was set in the exponential phase of the PCR, and the $\Delta \Delta^{{C_{\text{t}} }}$ method was used for data analysis. *Beta*-2-*microglobulin* (*B*_2_*M*, Rn00560865_m1) was used as the reference gene.

### TNF-α Determination

The concentration of TNF-α in the culture medium was measured using an ELISA method (eBioscience) according to the manufacturer’s instructions. The plate was coated with capture antibody and incubated overnight at 4 °C. After the plate was washed, it was blocked at room temperature for 1 h. A 100 μl of each sample was transferred in duplicate to pre-coated 96-well ELISA plates along with TNF-α standards (0; 15.6; 31.2; 62.5; 125; 250; 500; and 1000 pg/ml) and incubated overnight at 4 °C. After a washing step, a biotinylated TNF-α antibody was added, and the samples were incubated for 1 h at room temperature. Next, streptavidin conjugated to horseradish peroxidase (HRP) was added for 30 min, and the colorimetric reaction was initiated by the addition of the chromogen TMB (3,3′,5,5′-tetramethylbenzidine). A spectrophotometer (MultiskanSpectrum, ThermoLabsystems) was used to measure the absorbance at a wavelength of 450 nm, while the background was measured at 570 nm. The concentration of TNF-α was calculated from a standard curve and expressed as pg/ml.

### NGF Determination

The concentration of NGF in the culture medium was measured using an ELISA method (Cloud-Clone Corp. USA, Uscn Life Science Inc) according to the manufacturer’s instructions. A 100-μl aliquot of each sample was transferred to 96-well ELISA plates that were pre-coated with a specific anti-NGF antibody in duplicate with NGF standards (0; 39; 78; 156; 312; 625; 1250; and 2500 pg/ml) and incubated for 2 h at 37 °C. After a washing step, a biotinylated NGF antibody was added, and the samples were incubated for 1 h at 37 °C. Next, streptavidin conjugated to horseradish peroxidase (HRP) was added for 30 min, the colorimetric reaction was initiated by the addition of the chromogen TMB. The absorbance was measured at a wavelength (*λ*) of 450 nm using a spectrophotometer (MultiskanSpectrum, ThermoLabsystems). The concentration of NGF was calculated from a standard curve and expressed as pg/ml.

### BDNF Determination

The concentration of BDNF in the culture medium was measured using an ELISA method (RayBio USA, RayBiotech, Inc) according to the manufacturer’s instructions. A 100-μl aliquot of each sample was transferred to pre-coated 96-well ELISA plates in duplicate with BDNF standards (0; 12.29; 30.72; 76.80; 192; 480; 1200; and 3000 pg/ml) and incubated overnight at 4 °C. After a washing step, a biotinylated BDNF antibody was added, and the samples were incubated for 1 h at room temperature. Next, streptavidin conjugated to horseradish peroxidase (HRP) was added for 45 min, and the colorimetric reaction was initiated by the addition of the chromogen TMB. The absorbance was measured at a wavelength (*λ*) of 450 nm using a spectrophotometer (MultiskanSpectrum, ThermoLabsystems). The concentration of BDNF was calculated from a standard curve and expressed as pg/ml.

### Statistical Analysis

The data from all of the experimental groups are presented as the mean ± SEM. The results were analyzed using the STATISTICA 10 program. The homogeneity of the variance was analyzed with a Levene’s test. The subsequent statistical analyses used factorial analyses of variance (ANOVA) to determine the effects of two factors (factor 1-prenatal stress; factor 2-stimulants). Next, Tukey’s post hoc tests were applied. The level of significance was set at *α* = 0.05.

## Results

### The Effect of 24-h Corticosterone and Glutamate Exposure on Hippocampal Cell Damage

**Fig. 1:**
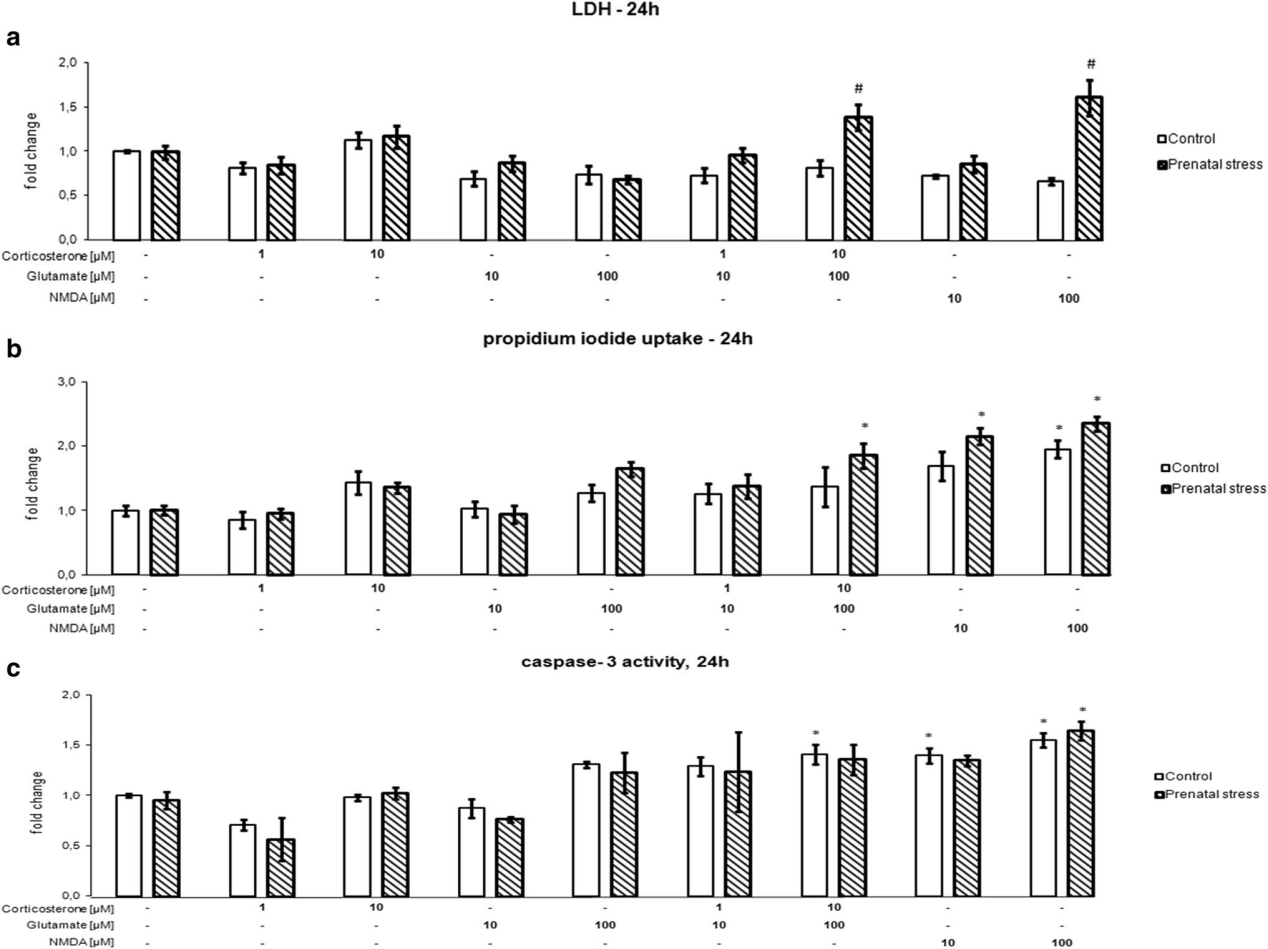
The effect of 24 h of exposure of hippocampal organotypic cultures to corticosterone, glutamate, corticosterone with glutamate, and NMDA on LDH release (**a**), propidium iodide uptake (**b**), and caspase-3 activity (**c**). The results are shown as a fold change relative to control cultures exposed to the appropriate vehicle and are expressed as the mean ± SEM. The significance of differences between the means was evaluated by Tukey’s post hoc tests following a factorial analysis of variance (ANOVA). *p < 0.05 versus control cultures; ^#^ *p* < 0.05 versus equally treated cultures from prenatally stressed rats; *n* = 8

The effects of 24 h of exposure of organotypic hippocampal cultures to corticosterone, glutamate, corticosterone with glutamate, and NMDA on LDH release (a), propidium iodide uptake (b), and caspase-3 activity (c) are shown in Fig. 1. After 24 h, an effect of the stimulants on LDH, PI, and caspase-3 was observed (LDH [*F*_8,75_ = 3.996, *p* < 0.05], PI [*F*_8,98_ = 7.457, *p* < 0.05], caspase 3 [*F*_8,53_ = 26.465, *p* < 0.05]), although an interaction between prenatal stress and the stimulants was noted only in the case of LDH (*F*_8,75_ = 3.730, *p* < 0.05). Additionally, an impact of prenatal stress on LDH and PI was observed (LDH [*F*_1,75_ = 16.398, *p* < 0,05], PI [*F*_1,98_ = 16.939, *p* < 0.05]). The presence of corticosterone or glutamate for 24 h at both the tested concentrations did not induce cell death, as determined by the release of LDH from necrotic cells into the medium, in neither the cultures of the control hippocampus nor those derived from prenatally stressed animals. When added separately, these compounds also had no effect on propidium iodide uptake or caspase-3 activity. LDH release and PI uptake were used to assess the cytotoxicity of the test compounds, whereas the LDH assay was used to measure necrosis and PI fluorescence was used to assess necrotic and end-stage apoptotic cells (Wolbergs et al. 2004). In contrast to these assays, caspase-3 is an executive apoptotic enzyme, and changes in its activity characterize apoptosis. Additionally, the application of corticosterone and glutamate at the lower concentrations did not exert an adverse effect on the viability of the cells in any of the three assays used. Only the addition of 10 μM corticosterone together with 100 μM glutamate enhanced LDH release and PI uptake, but this effect was observed only in the hippocampal cultures from prenatally stressed rats. Regarding LDH release, high concentrations of corticosterone and glutamate caused a statistically significant increase in this enzyme’s activity in the hippocampal cultures from prenatally stressed animals compared with tissue derived from control rats. The combined application of these compounds at high concentrations also increased the activity of caspase-3, but this change reached statistical significance only in the case of hippocampal cultures from control animals. At a concentration of 100 µM, NMDA, which was used in the present study as a positive control, potentiated the release of LDH from the hippocampus of prenatally stressed animals. At a concentration of 100 µM, this compound enhanced PI uptake in tissue from both control and stressed rats, while at a concentration of 10 µM, it enhanced PI uptake only in the stressed group. Caspase-3 activity was significantly increased by 100 µM NMDA in both examined groups, while 10 µM NMDA affected only hippocampal cultures from control rats.

### The Effect of 72-h Corticosterone and Glutamate Exposure on Hippocampal Cell Damage

**Fig. 2:**
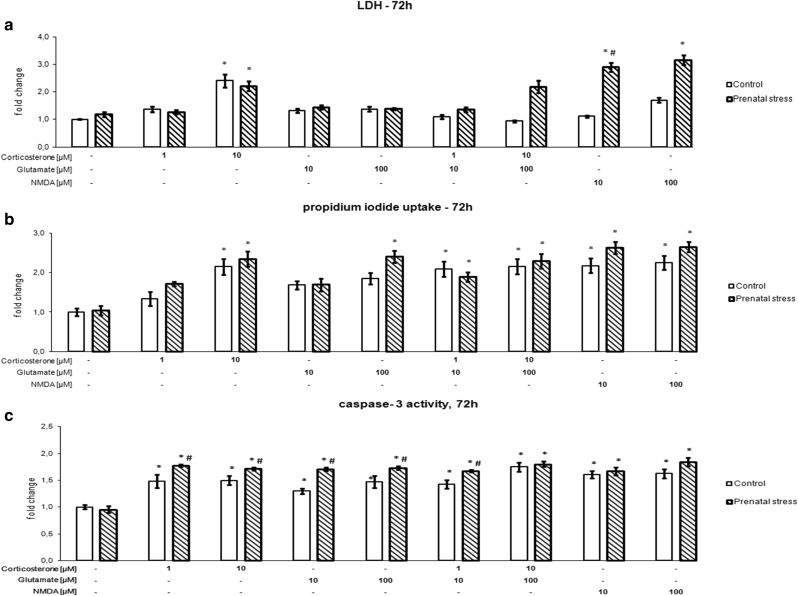
The effect of 72 h of exposure of hippocampal organotypic cultures to corticosterone, glutamate, corticosterone with glutamate, and NMDA on LDH release (**a**), propidium iodide uptake (**b**), and caspase-3 activity (**c**). The results are shown as a fold change relative to control cultures exposed to the appropriate vehicle and are expressed as the mean ± SEM. The significance of differences between the means was evaluated by Tukey’s post hoc tests following a factorial analysis of variance (ANOVA). *p < 0.05 versus control cultures; ^#^ *p* < 0.05 versus equally treated cultures from prenatally stressed rats; *n* = 8

**Fig. 3:**
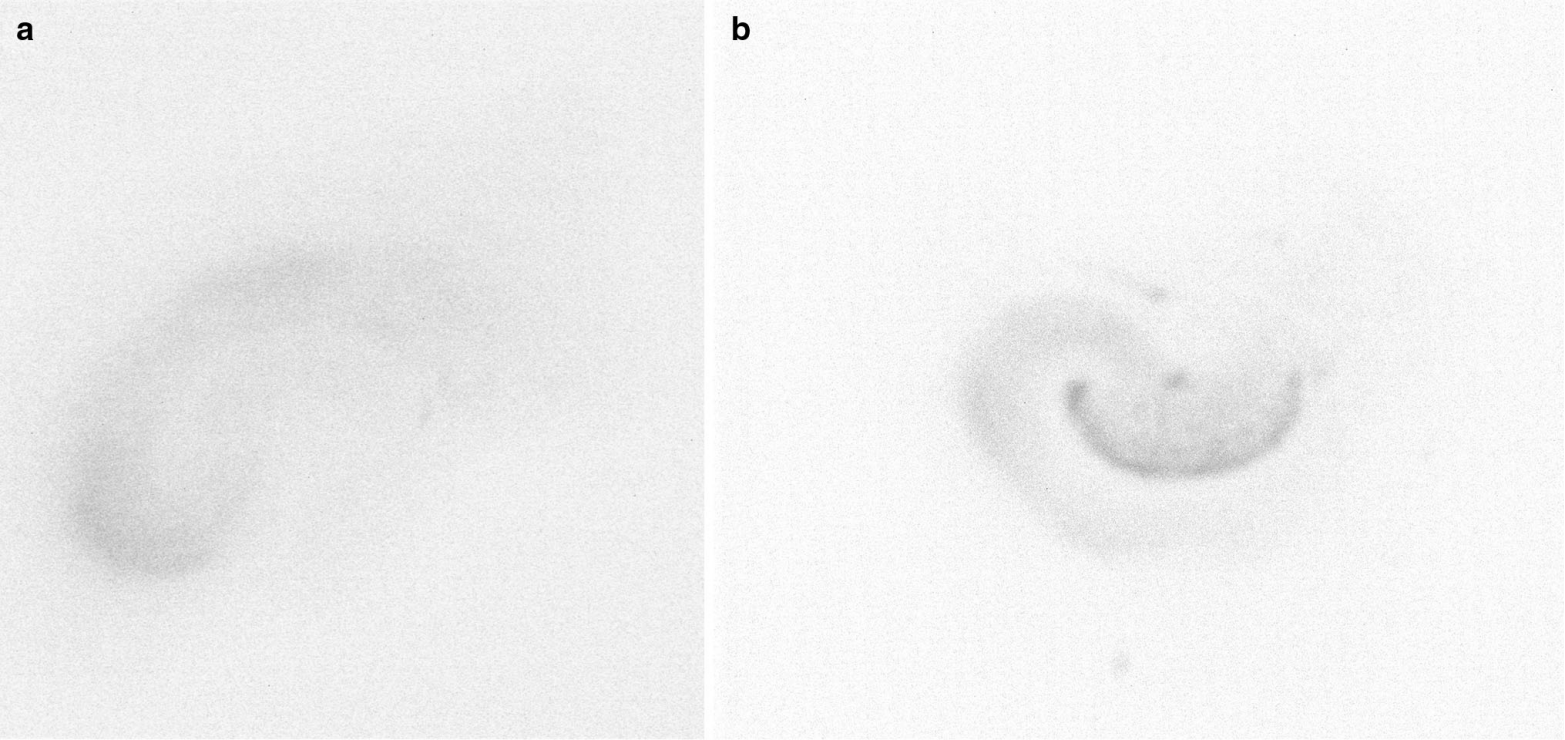
Representative images of propidium iodide uptake in vehicle-only treated hippocampal organotypic culture (**a**) and slices treated for 72 h with 10 µM corticosterone (**b**)

The effects of 72 h of exposure of organotypic hippocampal cultures to corticosterone, glutamate, corticosterone with glutamate, and NMDA on LDH release (a), propidium iodide uptake (b), and caspase-3 activity (c) are shown in Fig. 2. After 72 h, an effect of the stimulants on LDH, PI, and caspase-3 was noted (LDH [*F*_8,75_ = 7.3251, *p* < 0.05], PI [*F*_8,157_ = 6.896, *p* < 0.05], caspase 3 [*F*_8,58_ = 270.86, *p* < 0.05]), and an interaction between prenatal stress and the stimulants was observed for LDH and caspase-3 (LDH [*F*_8,75_ = 4.0687, *p* < 0.05], caspase-3 [*F*_8,58_ = 23.99, *p* < 0.05]). Additionally, an impact of prenatal stress on LDH, PI, and caspase-3 was observed (LDH [*F*_1,75_ = 19.1763, *p* < 0,05], PI [*F*_1,157_ = 15.015, *p* < 0.05], caspase 3 [*F*_1,58_ = 242.85, *p* < 0.05]). If the exposure to corticosterone and glutamate was prolonged from 1 to 3 days, a much greater degree of cell damage was observed. LDH release and PI uptake were potently and statistically significantly elevated by 10 µM corticosterone in hippocampal cultures from both control and prenatally stressed rats. Hippocampal tissues cultured in the presence of the solvent, showed almost no damage, determined by PI fluorescence (Fig. 3a), while the intensity of PI uptake was significantly higher in cultures treated for 72 h with 10 µM corticosterone (Fig. 3b). Glutamate had no effect on LDH release, but at a concentration of 100 µM, it enhanced PI uptake in the hippocampal cultures from prenatally stressed animals. The simultaneous addition of corticosterone and glutamate at both concentrations did not change LDH release but significantly enhanced the uptake of PI in the hippocampal cultures from control animals and those subjected to prenatal stress. At both concentrations tested, NMDA potentiated cell death, although a change was observed only in the hippocampal cultures from stressed animals in the case of LDH release, whereas PI uptake was increased in tissues from both groups. In contrast to the necrotic changes, an increase in the activity of the key apoptotic enzyme was observed in the hippocampal cultures derived from control and prenatally stressed animals following treatment with corticosterone, glutamate, their combination and NMDA. Moreover, corticosterone, glutamate, and the combination of the lower doses of corticosterone with glutamate increased caspase-3 activity statistically significantly and more strongly in the hippocampal cultures from prenatally stressed rats compared to those from control animals.

### The Effect of Corticosterone and Glutamate on the mRNA Expression of Pro-apoptotic Bax

**Fig. 4:**
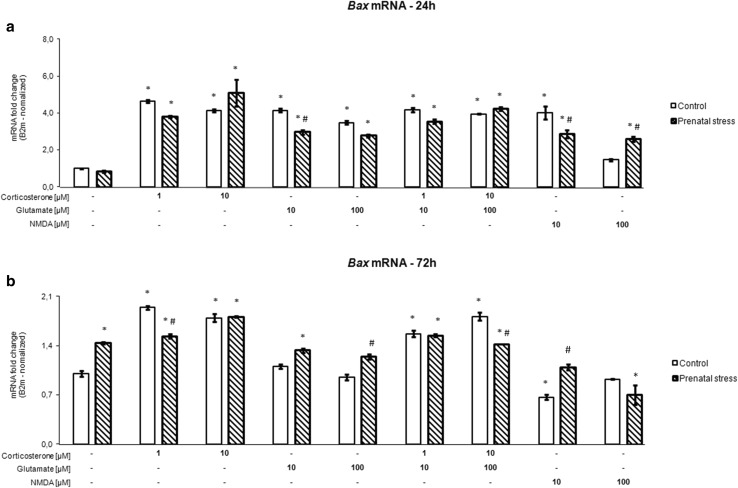
The effect of 24 h (**a**) and 72 h (**b**) of exposure of hippocampal organotypic cultures to corticosterone, glutamate, corticosterone with glutamate, and NMDA on Bax transcript levels. The results are shown as a fold change relative to control cultures exposed to the appropriate vehicle and are expressed as the mean ± SEM. The significance of differences between the means was evaluated by Tukey’s post hoc tests following a factorial analysis of variance (ANOVA). *p < 0.05 versus control cultures; ^#^ *p* < 0.05 versus equally treated cultures from prenatally stressed rats; *n* = 8

After 24 h (Fig. 4a) and 72 h (Fig. 4b), an effect of the stimulants and an interaction between prenatal stress and the stimulants was observed (24 h [*F*_8,102_ = 73.019, *p* < 0.05], [*F*_8,102_ = 10.572, *p* < 0.05], 72 h [*F*_8,106_ = 80.70, *p* < 0.05], [*F*_8,106_ = 17.28, *p* < 0.05]). At all tested concentrations, the presence of corticosterone, glutamate, and corticosterone together with glutamate for 24 h significantly enhanced the expression of mRNA for the main pro-apoptotic protein Bax in hippocampal slices from control and prenatally stressed rats (*F*_1,102_ = 8.684, *p* < 0.05). At 10 µM, NMDA also increased the expression of *Bax* mRNA in both groups, while 100 µM NMDA exerted such an effect only in tissue from prenatally stressed animals. In the 72-h cultures, corticosterone alone and added with glutamate evoked a significant rise in *Bax* mRNA levels, while glutamate induced an increase in expression of this factor only in slices from rats subjected to prenatal stress. In contrast, 10 µM NMDA inhibited *Bax* mRNA expression in the control groups, while 100 µM NMDA did so in prenatally stressed animals.

### Time-Dependent Effect of Corticosterone and Glutamate on Bdnf and Ngf mRNA Expression

**Fig. 5:**
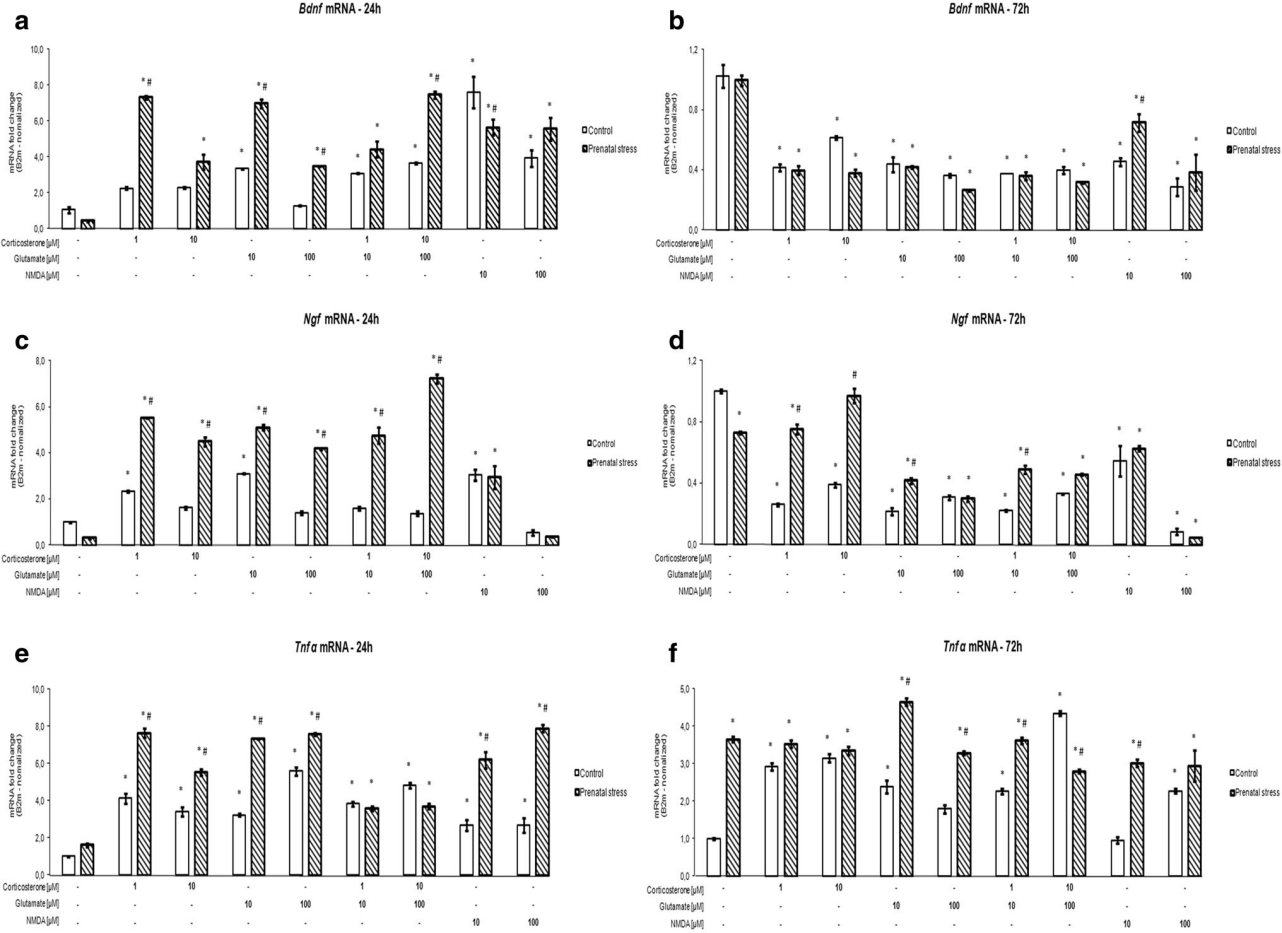
The effect of 24 h (**a**, **c**, **e**) and 72 h (**b**, **d**, **f**) of exposure of hippocampal organotypic cultures to corticosterone, glutamate, corticosterone with glutamate, and NMDA on transcript levels of *Bdnf* (**a**, **b**), *Ngf* (**c**, **d**), and *Tnf-*α (**e**, **f**). The results are shown as a fold change relative to control cultures exposed to the appropriate vehicle and are expressed as the mean ± SEM. The significance of differences between the means was evaluated by Tukey’s post hoc test following a factorial analysis of variance (ANOVA). *p < 0.05 versus control cultures; ^#^ *p* < 0.05 versus equally treated cultures from prenatally stressed rats; *n* = 8

After 24 h (Fig. 5a, c) and 72 h (Fig. 5b, d), an effect of the stimulants on *Bdnf* and *Ngf* mRNA expression was observed (*Bdnf* 24 h [*F*_8,94_ = 48.853, *p* < 0.05], *Ngf* 24 h [*F*_8,100_ = 98.082, *p* < 0.05], *Bdnf* 72 h [*F*_8,94_ = 37.162, *p* < 0.05], *Ngf* 72 h [*F*_8,100_ = 89.905, *p* < 0.05]), and an interaction between prenatal stress and the stimulants (*Bdnf* 24 h [*F*_8,94_ = 17.767, *p* < 0.05], *Ngf* 24 h [*F*_8,100_ = 55.304, *p* < 0.05], *Bdnf* 72 h [*F*_8,94_ = 3.598, *p* < 0.05], *Ngf* 72 h [*F*_8,100_ = 29.659, *p* < 0.05]) was noted. Additionally, an impact of prenatal stress on *Bdnf* (after 24 h of stimulation) and *Ngf* (after 24 and 72 h of stimulation) mRNA expression was observed (*Bdnf* 24 h [*F*_1,94_ = 114.808, *p* < 0.05], *Ngf* 24 h [*F*_1,100_ = 531.637, *p* < 0.05], *Ngf* 72 h [*F*_1,100_ = 98.386, *p* < 0.05]). The 24-h presence of corticosterone at both concentrations and of 100 μM glutamate increased mRNA levels of *Bdnf* but only in the hippocampal cultures from prenatally stressed rats, whereas 10 μM glutamate, corticosterone together with glutamate and NMDA caused such an effect in tissue from both stressed and control animals. The action of 1 μM corticosterone, 10 and 100 μM glutamate, and 10 μM corticosterone with 100 μM glutamate was significantly stronger in the hippocampal cultures from prenatally stressed compared to control animals. In contrast to the increase in *Bdnf* mRNA observed after 24 h, all investigated factors significantly reduced this parameter after 72 h. Similar time-dependent changes were also observed in the mRNA level of the other of the investigated growth factors, i.e., *Ngf*. Also, 24-h exposure to corticosterone, glutamate, and their combination enhanced the levels of *Ngf* mRNA in the hippocampal cultures from prenatally stressed rats, whereas in the control group, only 1 μM corticosterone and 10 μM glutamate exerted such an effect. NMDA caused a similar effect in both groups, but a significant increase was observed only for the lower concentration. After 72 h, a significant decrease in *Ngf* mRNA was observed in all investigated cultures, with the exception of samples containing hippocampal slices from prenatally stressed animals incubated with 10 μM corticosterone. The basal level of *Ngf* mRNA in samples from stressed animals was lower than that in control slices, but this difference was statistically significant only in the 72-h cultures.

### The Effect of Corticosterone and Glutamate on Tnf-α mRNA Expression

In contrast to the trophic factors whose mRNA expression increased within a short time after the addition of corticosterone or glutamate and then decreased after 72 h, the level of *Tnf-*α, a pro-inflammatory cytokine involved in neurodegeneration, increased under the influence of the examined factors at both times. After 24 h (Fig. 5e) and 72 h (Fig. 5f), an effect of the stimulants and of prenatal stress and an interaction between prenatal stress and the stimulants on *Tnf-*α mRNA expression was observed (24 h [*F*_8,94_ = 76.785, *F*_1,94_ = 360.666, *F*_8,94_ = 36.359, *p* < 0.05], 72 h [*F*_8,103_ = 22.126, *F*_1,103_ = 194.140, *F*_8,103_ = 28.623, *p* < 0.05]). After 24 h, corticosterone, glutamate, and NMDA more strongly increased the level of *Tnf-α* mRNA in the hippocampal cultures from prenatally stressed than in those from control rats. In the case of the 72-h incubation, the effect of glutamate, glutamate with corticosterone at the lower concentrations and 10 µM NMDA, but not of corticosterone alone, was stronger in tissue from stressed rats. The level of *Tnf-α* mRNA tended to increase in the hippocampal cultures from prenatally stressed rats that were cultured for 24 h, whereas after 72 h, this parameter was strongly and significantly increased.

### The Effect of Corticosterone and Glutamate on BDNF, NGF, and TNF-α Concentrations in the Culture Medium

**Fig. 6:**
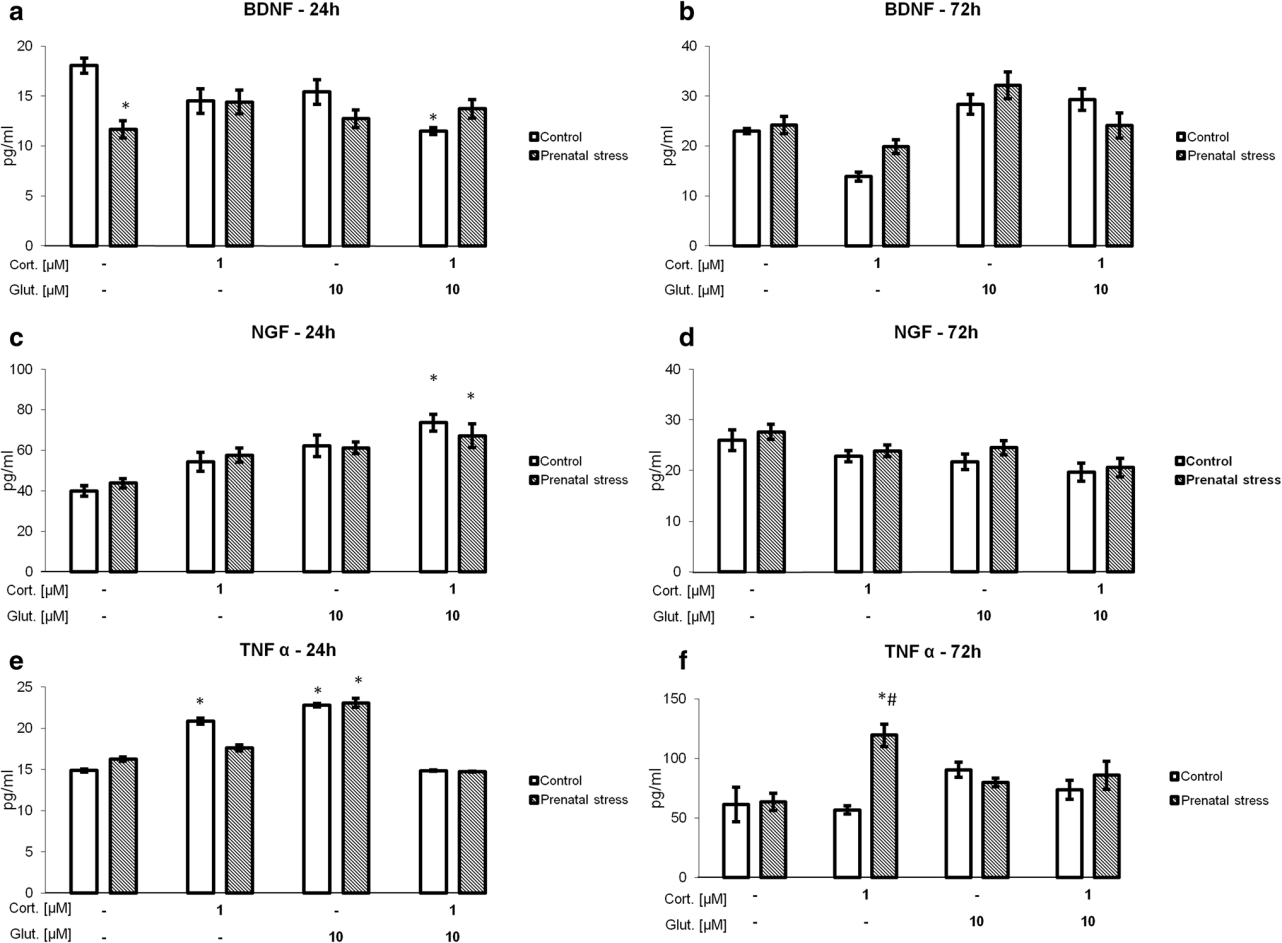
The effect of 24 h (a, **c**, **e**) and 72 h (**b**, **d**, **f**) of exposure of hippocampal organotypic cultures to corticosterone, glutamate, and corticosterone with glutamate on BDNF (**a**, **b**), NGF (**c**, **d**), and TNF-α (**e**, **f**) concentrations (pg/ml). The results are shown as the mean ± SEM. The significance of differences between the means was evaluated by Tukey’s post hoc tests following a factorial analysis of variance (ANOVA). *Cort.* corticosterone, *Glut*. glutamate, *p < 0.05 versus control cultures; *n* = 8

To test whether the observed changes in the levels of *Bdnf*, *Ngf*, and *Tnf-α* mRNA translated into appropriate protein levels, the concentrations of these factors in the culture medium from hippocampal slices incubated with physiologically relevant concentrations of corticosterone, glutamate, and their combination were determined by ELISA. After 24 h (Fig. 6a, c, e), an effect on the NGF and TNF-α concentrations in the culture medium was noted (*F*3,43 = 12.473, *F*3,49 = 22.818, *p* < 0.05) and after 72 h (Fig. 6b, d, f), an effect of the stimulants on BDNF, NGF, and TNF-α concentrations in the culture medium was observed (*F*_3,49_ = 17.641, *F*_3,48_ = 5.978, *F*_3,40_ = 6.8605, *p* < 0.05). An effect of prenatal stress on the level of TNF-α was also observed after 72 h of incubation (*F*_1,40_ = 7.4041, *p* < 0.05). An interaction between prenatal stress and the stimulants on the BDNF concentration in the culture medium was observed after 24 h and 72 h (*F*_3,41_ = 5.539, *F*_3,49_ = 2.950, *p* < 0.05). This effect was also observed for TNF-α after 72 h (*F*_3,40_ = 6.6054, *p* < 0.05). It was found that the BDNF level was lower in the 24-h hippocampal cultures from prenatally stressed compared to those from control rats (*F*_1,41_ = 5.022, *p* < 0.05). The BDNF level was also lower in control hippocampal slices incubated with corticosterone together with glutamate. However, in the 72-h cultures, neither prenatal stress nor any of the tested factors changed the BDNF level. NGF levels were significantly elevated in the 24-h hippocampal cultures from control and prenatally stressed rats, but only in samples that were treated with corticosterone together with glutamate. As in the case of BDNF, there were no changes in the level of NGF in the 72-h cultures. In the 24-h cultures, corticosterone and glutamate significantly increased the TNF-α level in hippocampal cultures from control animals, while in cultures from prenatally stressed rats, only glutamate exerted such effect. Joint application of corticosterone and glutamate had no effect on TNF-α release from the hippocampal slices into the culture medium in any of the groups. At 72 h of culture, only 1 μM corticosterone significantly increased the TNF-α level, but this effect was observed solely in the hippocampal cultures from prenatally stressed rats.

## Discussion

The present study indicates that the effect of corticosterone on hippocampal cell viability depends not only on the concentration of this hormone and its time of action but also on whether the animals were exposed to stress stimuli during the prenatal period. Unfavorable factors that act during this period change the sensitivity of the hippocampus, especially to the pro-apoptotic effect of corticosterone.

It is well known that glucocorticoids may have both neuroprotective and neurodegenerative effects and that their bidirectional action is mainly associated with the concentration and time of action of this hormone. In fact, a lack of corticosterone induces apoptosis in granule cells in the hippocampal dentate gyrus, while a slightly elevated concentration of corticosterone attenuates the neuronal injury induced by some neurotoxic substances, e.g., NMDA and beta-amyloid (Abrahám et al. 2000). Finally, at highly elevated concentrations, this hormone can cause nerve cell damage or aggravate the nerve cell damage induced by other factors (Diz-Chaves et al. 2013; Roy and Sapolsky 2003; Szczęsny et al. 2014). In the present study, high, potentially neurotoxic concentrations of this hormone were used. A corticosterone concentration of approximately 1 μM is considered to be similar to that observed during stress, and in fact, we previously observed a slightly lower concentration in the frontal cortex and hippocampus of rats subjected to prenatal stress and a slightly higher concentration after acute stress (Detka et al. 2014). Additionally, in other in vitro studies, including those involving organotypic cultures of the hippocampus, a similar concentration of corticosterone was used (Moosavi et al. 2008; Payne and Schurr 2001). At this concentration, the application of corticosterone, alone or jointly with glutamate, to hippocampal cultures for 1 and 3 days did not induce necrosis of hippocampal cells, as determined by LDH release. At this concentration, it also did not change PI uptake, although it potentiated the effects of glutamate in the 72-h cultures. A necrotic effect of corticosterone was observed only in the 72-h cultures when a very high 10 μM concentration of this hormone was applied. Our results concerning the influence of corticosterone on the necrotic process are consistent with previous studies. Moosavi et al. (2008) found that the addition of 1 μM corticosterone to the culture medium for 24 h had no effect on the viability of hippocampal neurons, whereas at higher concentrations, this hormone induced cell death. Corticosterone at this concentration and time of action also did not affect cell survival in mixed neuronal-glial hippocampal cultures or in hippocampal slice cultures (Roy and Sapolsky 2003).

In contrast to its effect on the necrotic process, in the 72-h cultures, the lower corticosterone concentration evoked a pronounced adverse effect on the activity of caspase-3, a key enzyme of apoptosis. This observation indicates that prolonged action of this hormone initiates the process of apoptosis rather than necrosis in the rat hippocampus. Moreover, corticosterone action on caspase-3 activity was significantly stronger in the hippocampal cultures from prenatally stressed compared to those from control rats. The induction of apoptosis by corticosterone was also confirmed by the increase in the expression of the pro-apoptotic protein *Bax*; this increase was already observed at low concentrations of this steroid, especially after 24 h of culture. Bax belongs to the Bcl-2 family, which includes many pro- and anti-apoptotic proteins that interact and regulate the formation of channels in the outer mitochondrial membrane and, as a consequence, affect the mitochondrial apoptotic pathway (Xiong et al. 2014). Among pro-apoptotic proteins, Bax exhibits the highest concentration in the brain (Pilchova et al. 2015). Bax may be an important element in the action of glucocorticoids, as it was shown that chronic stress or the administration of this steroid increased the levels of Bax oligomers in the rat cerebral cortex (Haack et al. 2008).

Although endogenous and exogenous glucocorticoids induce apoptosis in many peripheral tissues, to date, their effect on the apoptosis of nerve cells has been poorly examined. Roy and Sapolsky (2003) found that at a concentration of 1 μM, corticosterone acting for 24 h did not change the activity of caspase-3 in neuronal-glial hippocampal cultures, both when given alone and when potentiated the cytotoxic effects of kainic acid. Like in that study, in the present experiment, corticosterone at the same concentration had no effect on caspase-3 activity in 24-h cultures of hippocampal slices, while it strongly enhanced the activity of this apoptotic enzyme when it was present in the cultures for 72 h. Prior to the activation of caspase-3, an approximately fourfold increase in the expression of the pro-apoptotic protein Bax was observed. Bax is involved in earlier stages of apoptosis than caspase-3 (Tamatani et al. 1998), which explains the results showing a much greater increase in *Bax* mRNA expression in the 24-h compared to the 72-h cultures, while the increase in caspase-3 activity occurred after 72 h. Thus, the present study indicates that the adverse effects of corticosterone on the hippocampus are mainly related to the induction of apoptosis.

Assessment of the combined effect of the two main endogenous neurotoxic factors, i.e., glutamate and glucocorticoids, did not fully support the previous hypothesis. Many data indicate that glucocorticoids intensify the neurotoxic effects of many factors but themselves cause such an effect only in very high concentrations. For example, corticosterone has been found to increase neuronal damage in the hippocampus following acute hypoxia/ischemia, to augment the effect of hypoglycemia and to enhance the toxicity of beta-amyloid, glutamate, NMDA, and ethanol (Abraham et al. 2006). In particular, many studies have investigated the interactions between the neurotoxic effects of glucocorticoids and glutamate. In the present study, glutamate showed a cytotoxic effect in the 72-h model, as determined by PI uptake, and also had pro-apoptotic activity, but corticosterone potentiated only some of glutamate’s effects, primarily those caused by high, supraphysiological levels of glutamate, and only did so in prenatally stressed animals. Corticosterone did not increase glutamate’s effects on caspase-3 activity or on the concentrations of growth factors and the cytokine TNF-α; it even reduced TNF-α levels in the 72-h cultures of hippocampal slices from animals subjected to prenatal stress. These results suggest that the adverse effects of corticosterone and glutamate on brain cells may result from their effects on the same targets, but further studies are needed to verify this hypothesis.

The most important result of the present study was the demonstration that in physiological-like concentrations, the influence of corticosterone, glutamate, and both of these factors in combination on the activity of caspase-3 depended on whether the animals were exposed to stress stimuli during the prenatal period. These results provide evidence that stress during the last week of pregnancy in rats increases hippocampal sensitivity to the effects of these damaging compounds after birth. This finding supports our data and other previous data that elevated levels of glucocorticoids in the prenatal or first period of life act on brain development and produce many permanent changes, including increased reactivity of some brain tissues to damaging agents. Prenatal stress enhances glucocorticoid production in the mother and, as a consequence, evokes in the offspring HPA axis hyperactivity, sleep disturbances, reduced neurogenesis in the dentate gyrus of the hippocampus, cognitive deficits, and peripheral insulin resistance (Lemaire et al. 2006; Morley-Fletcher et al. 2004; Szymańska et al. 2009). Previously, we showed that prenatal stress also produced disturbances in brain glucose levels and metabolism in adult rats, but those changes were observed mainly in prenatally stressed animals under the influence of unfavorable factors later in life and not under basal conditions (Detka et al. 2014, 2015). As in the case of metabolic changes in the brain, the effect of corticosterone and glutamate was also stronger in the hippocampus from prenatally stressed than in control rats. One reason for this difference may be related to a permanently altered level of growth factors or pro-inflammatory cytokines. Indeed, considerable data indicate that chronic stress or glucocorticoid administration inhibits the synthesis of BDNF in the hippocampus. Glucocorticoids and stress also play an important role in the regulation of NGF synthesis, but data regarding the level of this factor in various models of stress remain controversial (Angelucci et al. 2003; Filho et al. 2015; Della et al. 2013). Current studies also show that glucocorticoids mediate the stress-induced activation of microglia and in this way enhance TNF-α secretion. In the prenatal stress model, an increase in *Tnf*-α expression and a decrease in *Bdnf* expression were observed in the hippocampus (Berry et al. 2015; Diz-Chaves et al. 2013). The present results demonstrate that a 24-h exposure to corticosterone and glutamate when applied separately and jointly increased *Bdnf*, *Ngf*, and *Tnf-α* expression, but a strong decrease in the expression of the neuroprotective factors was observed after 72 h, while expression of the neurodegenerative cytokine, *Tnf-α*, remained high. This result is consistent with other data showing that a short time after exposure to damaging factors, the levels of not only pro-inflammatory cytokines but also growth factors are raised, while after a longer time, a decrease in growth factor synthesis occurs. Among the factors we studied, i.e., TNF-α, BDNF, and NGF, only changes in the expression of TNF-α tend to be associated with increased apoptosis; however, the effect of corticosterone on the apoptotic process may arise from the effect of this hormone on other factors not examined in this study, including the inhibition of IGF-1 or a weakening of insulin receptor action (Trojan et al. 2016).

In summary, the present study showed the following: (1) corticosterone at a concentration of 1 μM evoked apoptotic but not necrotic processes in organotypic cultures of the hippocampus; (2) prenatal stress increased the pro-apoptotic effects of corticosterone and glutamate; (3) glutamate and corticosterone did not aggravate each other’s activity; and (4) increased TNF-α synthesis may be involved in the pro-apoptotic effects of corticosterone.

## Electronic supplementary material

Below is the link to the electronic supplementary material.

Supplementary material 1 (TIFF 923 kb)
