# Supplementary figures and images for: Pro-apoptotic Action of Corticosterone in Hippocampal Organotypic Cultures
Source: Neurotox Res. 2016 May 17;30:225–38. doi: 10.1007/s12640-016-9630-8 (PMC4947107; doi:10.1007/s12640-016-9630-8)

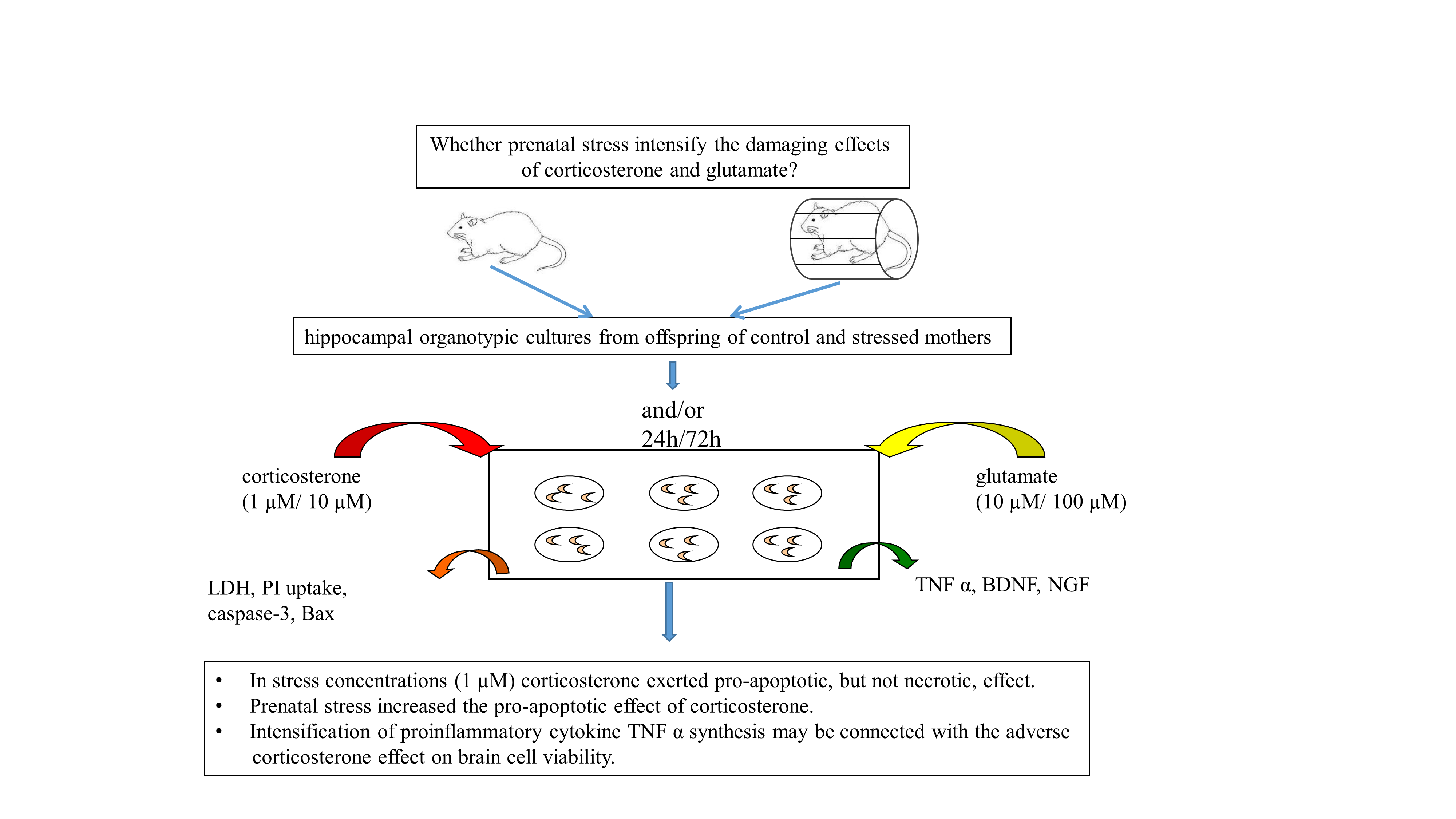

Supplement: Supplementary file 1 — Supplementary material 1 (TIFF 923 kb) [file 12640_2016_9630_MOESM1_ESM.tif]
